# Introducing Precision Addiction Management of Reward Deficiency Syndrome, the Construct That Underpins All Addictive Behaviors

**DOI:** 10.3389/fpsyt.2018.00548

**Published:** 2018-11-27

**Authors:** Kenneth Blum, Marjorie C. Gondré-Lewis, David Baron, Panayotis K. Thanos, Eric R. Braverman, Jennifer Neary, Igor Elman, Rajendra D. Badgaiyan

**Affiliations:** ^1^Graduate College of Biomedical Sciences, Western University of Health Sciences, Pomona, CA, United States; ^2^Department of Psychiatry, Boonshoft School of Medicine, Dayton VA Medical Center, Wright State University, Dayton, OH, United States; ^3^University of Vermont College of Medicine, Burlington, VM, United States; ^4^Division of Addictive Services, Dominion Diagnostics, LLC, North Kingston, RI, United States; ^5^Division of Precision Addiction Management, Geneus Health, LLC, San Antonio, TX, United States; ^6^Institute of Psychology, University of Eötvös Loránd, Budapest, Hungary; ^7^Department of Clinical Neurology, Path Foundation, New York, NY, United States; ^8^Division of Neuroscience and Addiction Therapy, Summit Estate Recovery Center, Los Gatos, CA, United States; ^9^Department of Neurogenetics Research and Addiction Therapy, The Florida House Experience, Deerfield Beach, FL, United States; ^10^National Human Genome Center, Howard University, Washington, DC, United States; ^11^Department of Anatomy, Howard University College of Medicine, Washington, DC, United States; ^12^Behavioral Neuropharmacology and Neuroimaging Laboratory on Addictions, Clinical and Research Institute on Addictions, Department of Pharmacology and Toxicology, Jacobs School of Medicine and Biomedical Sciences, University at Buffalo, Buffalo, NY, United States; ^13^Department of Psychology, University at Buffalo, Buffalo, NY, United States; ^14^Department of Psychiatry, Cooper Medical School of Rowan University, Camden, NJ, United States; ^15^Department of Psychiatry, Icahn School of Medicine at Mount Sinai, New York, NY, United States

**Keywords:** reward deficiency syndrome (RDS), dopamine homeostasis, genetic addiction risk score (GARS), comprehensive analysis of reported drugs (CARD), precision addiction management (PAM)

Worldwide daily, millions of people are unable to combat their frustrating and even fatal romance with getting high; for some, “high” may be just experiencing feelings of well-being. The neuroscience community conducts and funds, outstanding research using sophisticated neuroimaging and molecular-genetic applied technology to improve understanding of the complex functions of brain reward circuitry that has a key role in addiction symptomatology. While it is widely accepted that dopamine is a major neurotransmitter implicated in behavioral and substance addictions, there remains controversy about how to modulate dopamine clinically to treat and prevent various types of addictive disorders. A prudent approach may be biphasic; a short-term blockade followed by long-term dopaminergic upregulation. The goal of treatment would be to enhance brain reward functional connectivity volume, and target reward deficiency and the stress-like anti reward symptomatology of addiction. Such phenotypes can be characterized using the Genetic Addiction Risk Score (GARS)®. Dopamine homeostasis may thus be achieved via “Precision Addiction Management” (PAM)®, the customization of neuronutrient supplementation based on the GARS test result, along with a behavioral intervention.

## Introduction

Following almost three decades of genetic-based research related to identifying and characterizing addiction-related Reward Deficiency Syndrome (RDS), a paradigm shift is proposed in the prevention and treatment of all types of addictive behaviors. The novel adoption of “Precision Addiction Management” (PAM) based on genetic predisposition is imminent. Certainly, more research is necessary to further pinpoint the most appropriate candidate genes. However, an accelerated personalized, precision tool can be useful for the identification of highly convergent candidate gene single nucleotide polymorphisms (SNPs), associated in populations with RDS. Research directed toward improving treatment of substance use disorders in underserved populations is the basis of an NIH grant awarded to Drs. Kenneth Blum and Marjorie Gondré-Lewis. The research team is confident that eventually, the scientific community will seriously support new research directed toward the up-regulation of dopamine in mesolimbic structures with the goal of restoring homeostasis.

The interrelatedness of reward circuitry and the prefrontal cortices of the brain were not well-understood in the early sixties. The importance of the core neurotransmitters was not recognized. The functions of serotonin, GABA, dopamine, and acetylcholine were unknown, and endorphins were not even a part of our scientific acumen. The 1956 doctrine of Jellinek shocked the world when he proposed the concept of alcoholism as a disease, without much scientific evidence the idea was not generally accepted (1). However, most addiction scientists at that time agreed, in part, that deficiencies or imbalances in brain chemistry-perhaps genetic in origin –contributed to the cause of alcoholism.

## Reward deficiency syndrome (RDS)

In the early 70s, Blum investigated the theorized neurochemical mechanisms of some psychoactive drugs (alcohol and opioids) that had been observed initially in the work of Virginia Davis, Gerald Cohen, Michael Collins and others, as being related to the interface of alcohol and opioid use disorders (2–6). Following many other foundational studies from around the world, the Royal Society of Medicine published the RDS concept in 1996 (7). To date, there have been 150 articles in PUBMED, and the SAGE Encyclopedia of Abnormal and Clinical Psychology published a definition in 2017 (8). The RDS concept arose from the findings that dysfunction in the dopaminergic system are implicated in reward mechanisms in the brain and lead to substance seeking behavior and non-substance addictive behaviors (7) like pathological gambling (9–11), Tic Disorders (12, 13), Tourette's syndrome (14), and attention deficit hyperactivity disorder (ADHD) (15–17).

Mark Gold's “Dopamine Depletion Hypothesis,” proposed an important role for dopamine in the effects of cocaine. He observed that the development of chronic cocaine use disorder (CUD) was due to the euphoric properties of cocaine and followed the acute activation of central dopamine neurons. Overstimulation of these neurons and excessive synaptic metabolism is thought to result in dopamine depletion which may underlie dysphoric aspects of cocaine abstinence and cocaine urges (18). Neurochemical disruptions caused by cocaine are consistent with the concept of “physical” rather than “psychological” addiction (19). The proposal that followed this research was to treat CUD with dopamine agonist therapy. The powerful dopamine D2 agonist bromocriptine was found to reduce cocaine craving significantly after a single dose (20). The suggestion was that bromocriptine might be an effective, non-addictive pharmacological treatment for those with CUD and open trials indicated that low-dose bromocriptine might be useful in cocaine detoxification. In 1995, Lawford et al. administered bromocriptine or placebo to subjects with alcohol use disorder (AUD), in a double-blind study, they found that the most significant improvement in craving and anxiety occurred in the bromocriptine treated subjects with the Dopamine Receptor D2 (DRD2) A1 allele and attrition was highest in the placebo-treated A1 subjects (21). Unfortunately, we now know that chronic administration of this D2 agonist induces significant down-regulation of D2 receptors, thereby making it an ineffective deterrent to relapse and preventing its clinical use (22, 23).

## The cascade of reward neurotransmission

The Ventral Tegmental Area (VTA)-Nucleus Accumbens (NAC) pathway is part of a series of parallel integrated circuits within the “Brain Reward Cascade,” which involves the hypothalamus, dorsal raphe and substania nigra. The net release of dopamine is due to the interrelatedness of serotonergic, endorphinergic, endocannabinoidergic, GABAergic, glutaminergic, and dopaminergic neurotransmitter signaling. The VTA is the site of dopaminergic neurons, which tell the organism whether an environmental stimulus (like natural rewards, drugs of abuse, stress) is aversive or rewarding. While a less understood brain region, the pre-frontal cortex, including the anterior cingulate cortex and orbitofrontal cortex, provides executive control of choices such drug reinstatement as being pleasurable (reward). Most recently even the dorsal raphe which contains both serotonergic and glutaminergic neurons impact the GABA input at the substania nigra (24). Therefore, for reward processing while net dopamine released at the NAC (reward site) is key, it is impacted by many neurotransmitter systems. Neurotransmitter interaction at the mesolimbic brain region induce “reward” when dopamine well-known as an anti-stress and pleasure neurotransmitter is released from the neuron and interacts with a nucleus accumbens dopamine D2 receptor. Reward produced to maintain our drives is the consequence of a cascade of neurotransmission. Initially, the release of serotonin stimulates enkephalin, then, inhibits GABA at the substantia nigra and ventral tegmental area. GABA regulates the release of DA at the nucleus accumbens. Studies indicate that balancing dopamine, possibly via dopamine D2 agonists, especially when availability of this molecule, based on genes and even epigenetics, is compromised, has important therapeutic application which has been proposed by NIDA scientists (25).

A consensus of the literature suggests dysfunction in the brain reward cascade is caused by the genetic sequence variations that cause a hypodopaminergic trait. This trait leads to multiple drug-seeking behaviors; the brain of that person requires a dopamine fix to feel good. Reduced dopamine release causes individuals to crave dopamine and have a high risk for multiple addictive, impulsive and compulsive behaviors that have been shown release dopamine (26).

Non-drug behaviors like gambling (27) and high-risk drug use are reinforced by surges of dopamine activation in the nucleus accumbens via the D1 receptors of the direct striatal pathway and inhibition of the indirect corticostriatal pathway via D2 receptors. Chronic drug administration enhances the brain's reactivity to drug cues, reduces sensitivity to non-drug rewards and causes neuroplastic changes in glutamatergic inputs to the striatum and midbrain dopamine neurons. Self-regulation is weakened, and sensitivity to stressful stimuli and dysphoria is increased. These long-lasting drug-induced impairments call for interventions designed to mitigate and if possible reverse them (28).

## The development of precision addiction management

Blum et al. proposed that KB220Z; a mild neuro-nutrient formulation, can stimulate the D2 receptor (29). Blum's group advocates instigating dopamine release, to cause the induction of D2-directed mRNA to direct the proliferation of D2 receptors in the brain (30). For example, DNA-directed compensatory overexpression of the DRD2 receptors (a form of gene therapy), resulted in a significant reduction in alcohol craving behavior in alcohol-preferring rodents (31) and self-administration of cocaine (32). Thus, based on this model enhanced bioavailability of D2 receptors was shown to reduce craving.

Studies that showed rats with depleted neostriatal dopamine display increased sensitivity to dopamine agonists estimated to be 30–100 x in the 6-hydroxydopamine (6-OHDA) rotational model (33) were the basis for “denervation supersensitivity” (34). Denervation supersensitivity was identified as a putative physiological mechanism to help explain the enhanced sensitivity following intense acute dopaminergic D2 receptor activation in the face of hypodopaminergia. In contrast, promotion of long-term (chronic low vs. intense acute) dopaminergic activation by lower potency dopaminergic repletion therapy has been shown in clinical and neuro imaging studies, to be an effective modality when used to treat RDS behaviors including Substance Use Disorders (SUD), Attention Deficit Hyperactivity Disorder (ADHD), obesity and others, without side effects (35).

An unprecedented number of clinical studies validating this patented nutrigenomic technology for re-balancing brain chemistry, and optimizing dopamine sensitivity and function have been published. Here clinicians and neuroscientists are encouraged to continue to embrace the concept of “dopamine homeostasis” and search for safe, effective, validated and authentic means to achieve a lifetime of recovery, instead of reverting to anti-dopaminergic agents. Anti-dopaminergic agents are doomed to fail because chronic use continues and exacerbates hypodopaminergia while promoting powerful D2 agonists like bromocriptine and L-Dopa compromises needed balance (36). Increased resting state functional connectivity as well as an increased neuronal recruitment has been demonstrated acutely on fMRI in both animal and humans within 15 (animal) to 60 (human) minutes post administration of neuro nutrient therapy. These studies demonstrate neuronal dopamine firing in brain areas involved in reward processing and possible induced neuroplasticity and “dopamine homeostasis” (37, 38). The comprehensive role of dopamine as the mesolimbic system neurotransmitter underlying motivational function supports the low potency dopaminergic repletion therapy concept; sustainable, mild activation of D2 receptors (30).

## Precision addiction management (PAM)

The system is a holistic therapeutic model for treating RDS that includes the Genetic Addiction Risk Score (GARS) test for genetic risk predisposition and customization of neuronutrient supplementation to target the individual genetic allele variation, based on the GARS test results, and thereby deliver (PAM)® to patients.

**Figure 1:**
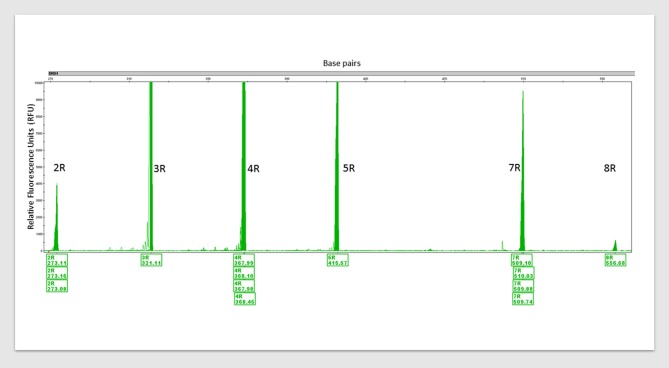
An example of PCR amplification of variants of dopamine receptor D4 (DRD4). *DRD4* (Dopamine Receptor 4) variants detected via polymerase chain reaction (PCR) amplification with multiple control samples. 2R to 8R = six different 48 base pair (bp) repeat sequences. 2R repeats = 48 bp twice, 3R = thrice and so forth. Peak height (y-axis) indicates fluorescence signal amplitude, peak location (x-axis) indicates fragment size (bp). Fragment sizes are shown below the peaks (base pairs). Humans carry two copies of this variant and their lengths are from 2R to 11R. Carrying one or both variants at 7R+ increases the risk of developing RDS. This is one of the eleven established risk variants assessed by the GARS test.

**Figure 2:**
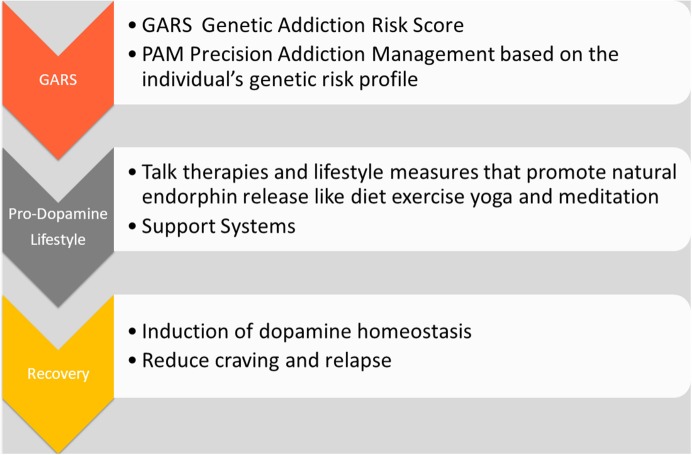
The Precision Addiction Management system. The schematic illustrates various elements related to “Precision Addiction Management (PAM)” and shows the interrelatedness of genetic testing, utilizing a patented Genetic Addiction Risk Score (GARS) and a customized polymorphic matched nutraceutical therapeutic adjunct KB220PAM.

See Figure 1 for an example of how simple genotyping for identified SNPs in individuals could identify targets for precision nutrigenomics treatment. Figure 1 shows the PCR amplification of four variants of dopamine receptor D4 (DRD4). Multiple repeats of DRD4 variants are associated with disorders within the RDS spectrum (39–41). In the figure, six different 48 bp repeat sequences are identified, from 2 repeats (2R) to 8R. The DRD4, DRD2, catechol-O-methyltransferase (COMT) are among genes within the mesolimbic reward pathway with SNPs that contribute to RDS, see Figure 2.

## The development of the genetic addiction risk score (GARS)

There are many examples of association studies involving genes and polymorphisms especially of the ten reward genes measured in the GARS test. Alleles of genes that affect the synthesis, degradation, reception, and transport of neurotransmitters (like enkephalin, serotonin, GABA, and dopamine) and enzymes like Monoamine Oxidase (MOA) A and COMT in the reward pathway of the brain were candidates for selection for the GARS test if they contributed to hypodopaminergia. Comings and Blum proposed that functional defects in the genes for these neurotransmitters result in dopamine deficit, later identified as RDS. They suggested that individuals with hypodopaminergia are at risk for seeking reward from RDS behaviors to satisfy their lack of natural rewards (42). Some examples of functional research and studies that associated RDS behaviors with the risk alleles of the genes and second messengers that comprise the GARS test follow.

## The characterization of phenotypes for the GARS

### The dopamine receptor DRD1 gene

The dopamine receptor DRD1 gene encodes the most abundant dopamine receptor in the central nervous system: the D1 subtype (43). While the D2, D3, and D4 receptor subtypes inhibit adenylyl cyclase activity, the D1 receptor stimulates a critical brain molecule adenylyl cyclase, which activates cyclic AMP (required for proper nerve brain function). The nucleotide guanidine (G) can be replaced by the adenine (A) and roughly a quarter of the human population carries one or two copies of this risk variant allele. Peer-reviewed studies involving the DRD1 gene show that carriers have an increased risk for many substance abuse and novelty seeking (NS) reward deficiency behaviors. In one of hundreds of association studies, Liu et al. looked at drug dependence and impulsive behavior and found that DRD1 gene polymorphisms are related to heroin dependence in a Chinese Han population, however, two of eight SNPs did not associate with impulsivity (44). Huang et al. found a significant association between the DRD1 and Nicotine Dependence (ND) (45). Recently they explored differential allelic expression of the DRD1 modulated by microRNA miR-504 (46). Other positive association studies on abusable drugs related to the D1 polymorphisms provide informative data indicating risk (47–50).

### The dopamine receptor DRD2 gene

The dopamine receptor DRD2 gene encodes the second (D2) subtype of the dopamine receptor. While the D1 receptor subtype activates, the D2 receptor inhibits adenylyl cyclase activity and reduces the intracellular concentration of cyclic AMP (51). DRD2 located near Ankyrin Repeat and Kinase Domain Containing 1 (ANKK1) at chromosome 11 q23.2, is involved in signal transduction (52). The ***Taq A1*** genetic variant in ANKK1 equates functionally to 30–40% lower density of dopamine D2 receptors resulting in hypodopaminergic function (53). The DRD2 risk variant of interest (rs1800497) is downstream at chr11:113400106 where the nucleotide guanine (G) replaces the adenine (A). In 1986 inspired by a reported unconfirmed genetic association in depressed Amish subjects, Blum et al. discovered the first genetic association with a behavior, severe alcoholism, foundational work within the field of behavioral genetics (54). Initially, **Taq 1A** (rs1800497) was thought to be within the DRD2 gene, but as sequencing technology advanced and the location was corrected to exon 8 of the nearby ANKK1 gene. This risk allele is found in approximately 100 million people in the USA, with the highest frequency in Native Americans (85%) and the lowest among Western European Jews (6%).

The first of few examples of association studies is one from Singh, Ghosh, and Saraswathy. They looked at the role of dopamine receptors in susceptibility to alcohol dependence (AD) concerning three sites of the *DRD2* gene (-141C Ins/Del, TaqIB, and TaqID) and ***TaqIA*** site of ANKK1 gene among Meiteis of Manipur, a Mendelian population of India, in association with AD. They found ANKK1 TaqIA polymorphism significantly associated with AD (odds ratio = 2.13, 95% confidential interval 1.04–4.39, *P* < 0.05), whereas a borderline significance of the −141C Del allele was observed (*P* = 0.059) (55).

Panduro et al. analyzed DRD2/ANKK1 genotyped a cross-section of 680 unrelated subjects including two Native Amerindians groups (87 Nahuas and 139 Huicholes), and two Mestizos groups (158 subjects from Tepic, Nayarit and 296 subjects from Guadalajara, Jalisco) by PCR-RFLP and allelic discrimination assays. Heavy drinking was considered ≥300 g alcohol/week. They concluded that the DRD2/ANKK1 A1 allele was present at a high frequency in Mexican populations and the A1/A1 genotype associated with heavy drinking in Mestizos. The highest frequencies of the A1 allele, exhibited worldwide to date were documented among the Native Amerindians (56).

Wang et al. investigated whether predisposing genetic variants and personality traits may be specific to a particular class of addiction or common to all addictions. They recruited 175 opiate-dependent patients, 102 alcohol-dependent patients, and 111 putative healthy controls diagnosed using DSM-IV criteria and assessed with Tridimensional Personality Questionnaire (TPQ). They genotyped dopamine D2 receptor (DRD2), 5-HTT-linked promoter region (5-HTTLPR), and aldehyde dehydrogenase 2 (ALDH2) genes using Polymerase Chain Reaction (PCR). They concluded that both alcohol- and opiate-dependent patients, have common genetic variants in DRD2 and 5-HTTLPR but specific for ALDH2. They found higher NS and Harm Avoidance traits in both patient groups with the interaction with DRD2, 5-HTTLPR, and ALDH2 genes (57).

Merritt and Bachtell in their non-genetic physiological study looked for the expression of the D2 dopamine receptor subtype as predictive of D2 dopamine receptor function and cocaine sensitivity that would enable cocaine abuse in rats. Quinpirole [D2 dopamine receptor agonist] was used to classify rats as high (HD2) or low responders (LD2). Results demonstrated that HD2 rats have greater cocaine conditioned place preference, enhanced sensitivity to the locomotor stimulating properties of cocaine, and self-administer more cocaine compared to LD2 animals. These findings suggested that individual differences in D2 dopamine receptor sensitivity may be predictive of cocaine sensitivity and reward (58).

Persico, Bird, Gabbay, and Uhl tested the hypothesis that a DRD2 gene variant might be more prominent in polysubstance users who preferentially use psychostimulants than in addicts who prefer opiates and those with no drug preference. Their results were consistent with the hypothesis that DRD2 gene variants marked by Taq1 A1 and B1 polymorphisms may work, probably in concert with other genetic and environmental factors, to enhance vulnerability to psychostimulant abuse (59).

Noble et al. examined the allelic prevalence of the D2 dopamine receptor (DRD2) gene in male cocaine-dependent (CD) Caucasian (non-Hispanic) subjects to determine the relationship of DRD2 alleles to family history and selected behavioral measures. The data showed a strong association of the minor alleles (A1 and B1) of DRD2 with cocaine dependence and suggested that a gene, located on the q22-q23 region of chromosome 11, confers susceptibility to this drug disorder (19).

Wang et al. used the MassARRAY system to identify markers that contribute to the genetic susceptibility to heroin addiction. They compared 334 patients with heroin dependence, and 299 healthy controls who participated in the research. Their findings indicated a role for DRD2 polymorphism in heroin dependence in the Chinese Han population and may be helpful for future genetic or neurobiological studies on heroin dependence (60). Most recently, Zhang et al. (61) found a 35.8 kilobases haplotype spanning ANKK1 and DRD2 is associated with heroin dependence in Han Chinese males (61).

### The dopamine receptor DRD3 gene

The dopamine receptor DRD3 gene encodes the D3 subtype of the five (D1–D5) dopamine receptors. As with D2 receptors, the activated D3 receptor inhibits adenylyl cyclase DRD3. Having a cytosine (C) instead of thymine (T) intensifies the effect of dopamine, magnifying the “high” observed with alcohol and cocaine to dangerous levels (62). The location of this receptor is within the older, more emotionally bent, limbic areas of the brain, including the pituitary gland, the olfactory bulb (smell) and the nucleus accumbens (cravings, aversions, reward). This gene demonstrates increased risks for alcohol, cocaine, and heroin dependence as well as RDS behaviors including ADHD, OCD, and even pathological aggression.

Thome et al. investigated the distribution of a dopamine D3 receptor gene polymorphism (Ball) in patients suffering from AD and compared with non-dependent controls. The allele A1 occurred significantly more frequently among patients compared to controls. Patients with the genotype A1/A2 showed significantly higher defined NS scores in the tridimensional personality questionnaire (TPQ) than patients with the genotype A1/A1. While this seems counter intuitive it can be explained by heterosis a well-known genetic phenomenon extensively discussed in the literature. There were significantly more individuals with higher NS scores and fewer individuals with lower NS scores than expected. The results of this study support the hypothesis of a genetically determined involvement of the dopaminergic system in AD (63).

Another study by Huang et al. investigated 13 single nucleotide polymorphisms (SNPs) spanning a region of the dopamine D(3) receptor gene (DRD3) to determine whether DRD3 is associated with ND. They studied a set of 2,037 subjects in 602 nuclear families representing two distinct American populations using three ND measures, namely, smoking quantity (SQ), the Heaviness of Smoking Index (HSI), and the Fagerström Test for ND (FTND). The results indicate that DRD3 associated significantly with ND in the European American cohort, and that rs6280, a functional polymorphism causing an amino acid change of serine to glycine (Ser9Gly) in the N-terminal extracellular domain of the D(3) receptor, likely is causative of the association between DRD3 and ND (64). Other positive association studies involving avoidant and obsessive personality traits and disorders, violent behavior and response to morphine reveal the importance of DRD3 polymorphisms in RDS (65–67).

### The dopamine receptor DRD4 gene

The dopamine receptor DRD4 gene that encodes for the receptor (subtype) D4. Dopamine receptors are responsible for neuronal signaling in the old reptilian mesolimbic system of the brain, which is an area that regulates emotions as well as RDS addictive behaviors. The DRD4 located on chromosome 11 at 11p15.5., is a G-protein receptor which is activated by the chemical messenger dopamine. Two different variants are measured in the GARS test. The first is a SNP located at rs1800955, −521 C>T and the risk allele is C. The second, is a Variable Number of Tandem Repeats (VNTR) located in intron 3, 48. The base–pair repeat; <7 is short, while if there are >7R, the risk allele is long VNTR equal or >7–11 repeats (68, 69). There are hundreds of studies of the DRD4 gene, and many of these studies have linked these risk variants to neurological and psychiatric conditions including schizophrenia, bipolar disorder, anhedonia, ADHD, Addictive behaviors, Parkinson's disease, eating disorders, and even anorexia nervosa (a non-eating repetitive RDS behavior). The underlying brain mechanism is D2-like in which the activated receptor inhibits the enzyme adenylate cyclase, thereby reducing the intracellular concentration of the second messenger cyclic AMP (required for cell function). The 7R allele appears to have been selected, for providing a survival advantage, about 40,000 years ago. It has been shown that compared to sedentary populations, the frequency of the 7R variant of *DRD4*, is much higher in nomadic populations suggesting its association with modern day “NS.” One important feature is that good parenting (epigenetic) was associated with appropriate decision making even at the age of four. Thus, early knowledge of this 7R or greater risk variant could be very helpful to prevent risk for substance use: opiate, alcohol, cannabis, glucose, and nicotine as well non-substance RDS behaviors: ADHD, Novelty seeking, Conduct Disorder (CD), hypersexuality, pathological aggression, and others.

Multiple repeats of DRD4 variants are associated with disorders within the RDS spectrum. Gervasini et al. conducted research to determine the effect of functional polymorphisms and haplotypes of the DRD4 gene on general psychopathological symptoms of 273 eating disorder (ED) patients [199 with Anorexia Nervosa (AN) and 74 with Bulimia Nervosa (BN)] who completed the SCL-90R symptom inventory. They found that certain combinations of DRD4 variants haplotype ^*^2 (non7R-TR long-C-C) associated with higher scores in the three global SCL-90R indices the Global Severity Index (GSI), the Positive Symptom Distress Index, (PSDI), and the Positive Symptom Total (PST) after Bonferroni correction (*p* ≤ 0.01 in all instances). They also found that these polymorphisms may contribute to psychopathological features like Somatization, Obsessive-Compulsive, Anxiety, Phobic anxiety, Paranoid ideation, in BN patients (41).

In another example Dragan and Oniszczenko analyzed the association between the variable number tandem repeat (VNTR) DRD4 exon III polymorphism and intensity of PTSD symptoms in 107 (57 women and 50 men) survivors of a flood aged 14–62. PTSD symptoms were more intense for participants with at least one copy of the long (seven or eight repetitions) DRD4 allele than participants who did not have these alleles (40). Huang et al. used a transmission disequilibrium test of DRD4 exon III 48 bp variant-number-tandem-repeat polymorphism and tic disorder. Their results revealed an association between the longer alleles of DRD4 exon III 48 bp VNTR polymorphism and tic disorder accompanied with ADHD, thus suggesting a possible genetic risk factor of tic disorder with ADHD in Chinese (39). One interesting recent study by Ji et al. (70) involving epigenetics revealed that DNA methylation of *DRD4* may be responsible for the pathophysiology of drug addiction (70).

### The dopamine transporter gene

The dopamine transporter gene (DAT1/SLC6A3) gives instructions for the production of a membrane-spanning protein that controls the reuptake (recycling) of dopamine from the synapse. The dopamine transporter helps regulate the level of neurotransmitter present in the synapse and controls how long a signal resulting from neurotransmitter release lasts (71).

The function of DAT1 is to clear excess dopamine released from the pre-neuron into the synapse and prevent uptake into the receptors on the next neuron. Much research, both biochemical and structural, has been performed to obtain clues about the mechanism of reuptake.The activity of clearing dopamine from the synapse is dependent on the variant form of this gene. So under normal conditions, the dopamine active transporter protein pumps the chemical messenger dopamine out of the synaptic cleft back into the cytosol of the pre-neuron cell. The DAT1 gene is located on chromosome 5 at p15. The gene has a variable number tandem repeats (VNTR) at the 3′end of the gene and another in the intron 8 region (72). The importance here is that differences in the VNTR, for example, 10R vs. 9R have been shown to effect the basal level of expression (activity) of the transporter. Indeed it has been demonstrated that the 9R is a risk form because it has a much higher ability to clear DA from the synaptic cleft compared to the 10R allele (73). Therefore, carriers of the 9R are more prone to both substance and non-substance RDS addictive behaviors due to hypodopaminergia (low dopamine function). The regional brain distribution of the DAT includes high dopamine-containing neurons in the old reptilian limbic system similar to the DRD2 receptor distribution (74). The maximum expression of the DAT1 gene is found in a parts of the brain called the substantia nigra and ventral tegmentum area (75) [brain regions containing large amounts of the inhibitory chemical messenger GABA that fine-tunes dopamine release at the reward site]. It is also interesting that DAT is co-localized with the D2 receptors (76).

There exists over 2,700 studies (PUBMED 7-14-18) concerning the role of the DAT1 gene and predisposition for the use of substances: particularly, heroin, alcohol, cocaine, and nicotine dependence; and non-substance RDS behaviors: ADHD, depression (Anhedonia), and PTSD are included.

Sullivan et al. focus on the role of aberrant dopaminergic signaling, interaction with dopamine transporter DAT, a cocaine target, and the dopamine D2 receptor in subjects and controls from the Miami Dade County Brain Bank splicing polymorphism rs2283265 of DRD2, encoding D2 receptors, was shown to confer risk (odds ratio ~3) of cocaine overdose/death. This risk was attributable to the minor allele of rs2283265 enhanced significantly in homozygous carriers of the main 6-repeat allele of DAT rs3836790to OR = 7.5 (*P* = 0.0008). In contrast, no significant risk to carriers of the minor 5-repeat DAT allele was demonstrated. The results demonstrated gene-gene-drug interaction affecting the risk of fatal cocaine intoxication (77).

Cinque et al. used transporter (DAT) knockout (KO) and heterozygous (HET) mice to investigate diseases with altered dopamine transmission such as attention-deficit/hyperactivity disorder (ADHD) and obsessive-compulsive disorder (OCD). These diseases characterized by poor decision making and executive function have been studied using many animal models. DAT KO rats appeared less sensitive to rewarding stimuli than wild-type (WT) and HET rats: they also showed a prominent hyperactive behavior with a rigid choice pattern and a wide number of compulsive stereotypies. Moreover, when the effects of amphetamine (AMPH) and RO-5203648 were tested, the AMPH accentuated impulsive behaviors in WT and HET rats, it did not affect in DAT KO rats (78).

### Monoamine oxidase a gene

Monoamine oxidase A, (MAO-A), an enzyme, is encoded by the MAOA gene in humans. Two neighboring gene family members MAOA and MAOB encode mitochondrial enzymes which catalyze the oxidative deamination (breaks down the molecules) of catecholamines, like dopamine, norepinephrine, and indoleamines like serotonin. This gene is associated with a variety of psychiatric disorders, including antisocial behavior. The MAOA gene located on only the X chromosome at Xp11.3, the short (p) arm of the X chromosome, at position 11.3. a crucial enzyme for healthy brain function degrades chemical messengers like dopamine and serotonin (79–82).

The risk variant is 4R found at the 3′ 30 base-pair Repeat (R) on X chromosome only. The 3.5R and 4R variants have been found to be more highly active than 3R or 5R. Carriers of the 3.5 and 4R may display hypodopaminergia (low dopamine function) whereby too much of dopamine is broken down in the presynaptic neuron which may result is less dopamine to be released into the synaptic cleft. The 4R has been associated with Alzheimer's disease, aggression, panic disorder, bipolar affective disorder, major depressive disorder, and ADHD. There are over 900 studies on the MAO-A gene showing risk for substance use disorder: alcohol, opioid, and nicotine dependence, as well as obesity, and RDS behaviors: harm avoidance, NS, and ADHD.

Regarding catabolism the studies reveal risk for MAOA, Wang et al. conducted a family-based association analysis of AD in the COGA sample in the Australian twin-family study and found support for an association of MAOA gene (*P* = 4.14 × 10(−4) for rs979606) and AD (83).

Ducci et al. investigated the interaction between a functional monoamine oxidase A (MAOA) locus and childhood sexual abuse (CSA). They tested whether MAOA-LPR influences the impact of CSA on alcoholism and antisocial personality disorder (ASPD) in a sample of 291 women, 50% of whom had experienced CSA. They also tested whether haplotypes covering the location of both MAOA and MAOB) genes predict risk for alcoholism and ASPD better than the MAOA-LPR locus alone. Three haplotypes showed the MAOA-LPR low activity allele. The most abundant was among alcoholics (*P* = 0.008) and antisocial alcoholics (*P* = 0.001). Independently from ASPD the MAOB haplotype associated significantly with alcoholism (*P* = 0.006), and antisocial alcoholism (*P* = 0.03) (84).

Tikkanen et al. found that the PCL-R symptom inventory total score predicts impulsive reconvictions among offenders with high-activity MAOA (6.8% risk increase for every one-point increase in PCL-R total score, *P* = 0.015) but not among offenders with low-activity MAOA. Antisocial behavior and attitudes were found to predict reconvictions in both high and low activity MAOA genotypes a17% risk increase and a 12.8% increase, respectively. In a meta-analysis, Yang et al. found an association between MAO gene polymorphisms and smoking behavior. The meta-analysis showed the T allele in MAO-A C1460T reduced the risk of heavy smoking (OR = 0.66, 95% CI: 0.52–0.84; I(2) = 0.0%), especially in Caucasians. However, the active group in MAO-A VNTR increased the likelihood of smoking cessation failure in males (OR = 1.49, 95% CI: 1.01–2.22; I(2) = 0.0%) and the A allele in MAO-B G644A reduced the risk of heavy smoking in males (OR = 0.20, 95% CI: 0.04–0.98) (85).

Gorodetsky et al. investigated the interactive effect of MAOA-LPR genotype and a history of childhood trauma in predicting aggressive behaviors in a population of 692 male prisoners. Within the group not exposed to physical neglect (PN), carriers of the MAOA-LPR high-activity variant were more aggressive: (tR = 2.47, *P* < 0.014). They observed a crossover effect in that the increase in aggression scores with PN was greater in low-activity individuals (tR = 5.55, *P* < 0.0001) than in high-activity individuals (tR = 4.18, *P* < 0.0001). These findings suggested that childhood trauma and the functional MAOA-LPR polymorphism may interact to specifically increase the risk for over-aggressive behavior but not impulsivity or hostility. The MAOA-LPR low-activity variant may be protective against the development of aggressive behavior under low-stress conditions (86).

Vanyukov et al. looked for evidence of an association of a dinucleotide repeat polymorphism at the MAOA gene with early onset alcoholism/substance abuse. They found a correlation between the presence/absence of the disorder and the length of the MAOCA-1 repeat was significant in males only, with both increased risk for the disorder and lower age of onset of substance abuse associated with “long” alleles (repeat length above 115 bp) (87).

Tikkanen et al. looked at the relationship between MAOA the effects of heavy drinking and childhood physical abuse (CPA) on risk for severe impulsive acts of violence among violent alcoholic offenders. The sample population of 174 male impulsive, alcoholic, Finnish, violent offenders assessed after 8 years of non-incarcerated follow-up mostly exhibited antisocial (ASPD) or borderline personality disorder (BPD) or both. Logistic regression analyses demonstrated that both CPA and heavy drinking were significant independent predictors of recidivism in violent behavior (OR 5.2, *p* = 0.004 and OR 5.3, *p* = 0.003) among offenders having the high MAOA activity genotype (MAOA-H), but these predictors showed no effect among offenders carrying the low MAOA activity genotype (MAOA-L) (88). The work of Huang et al. (89) suggested the interaction of DRD2 rs1079597 and MAOA rs309850 3-repeat affects smoking intensity in young Taiwanese men (89).

### The catechol-o-methyltransferase gene

The COMT gene (location 22q11.2) provides instructions for making an enzyme called COMT. Enzymes facilitate chemical reactions without the enzyme being changed (90). The gene makes two versions of COMT enzyme. The longer form, called membrane-bound catechol-O-methyltransferase (MB-COMT), is chiefly produced by nerve cells in the brain. The COMT enzyme destroys the dopamine molecule in the synapse and helps maintain appropriate levels of neurotransmitters (dopamine and norepinephrine) in the brain. In some people, there is a variation of a single protein building block (amino acid) in the enzyme (91). The amino acid valine (Val) replaces the amino acid methionine (Met), expressed as Val108/158Met. Carriers of two copies of MET have very heightened dopamine function because COMT activity is low and less dopamine is broken down, while two copies of Val, destroy more dopamine, and lower dopamine function (hypodopaminergia) is the result. The double copy (homozygote) of Val variant metabolizes dopamine at up to four times the rate of its Met counterpart. There have been over 2,400 studies involving the COMT gene. Val carriers are at increased risk for substance-related reward deficiency behaviors: including dependence on alcohol, cannabis, glucose; opiates/opioids, stimulants, and nicotine; or non-substance-related: ADHD, Oppositional Defiant Disorder, pathological aggression, panic disorder, anxiety, Obsessive-Compulsive Disorder (OCD) (92).

Hill et al. investigated caudate volume and working memory with brain scans and fMRI in offspring with childhood disorders followed until adolescence. They were tested for genotypic variation in the COMT and DRD2 genes. Caudate volume and working memory were reduced in association with externalizing disorders of childhood-adolescence and associated with variation in COMT and DRD2 genes (93).

Sery et al. performed a restriction analysis for the detection of the Val158Met polymorphism to look at the association between high-activity COMT allele and alcoholism in DNA samples from 799 subjects in total (279 male alcoholics and 120 female alcoholics, 151 male controls and 249 female controls). They found a significant difference between male alcoholics and male controls in allele and genotype frequencies (*p* < 0.007; and *p* < 0.04, respectively) (94).

Guillot et al. examined associations of COMT rs4680 with dimensionally and categorically measured gambling and drinking problems in a non-clinical sample (139 Caucasian adults). They found that COMT rs4680 was related to both dimensionally and categorically measured gambling and drinking problems and may be a genetic risk factor that contributes to the development of both problems 36 (95).

Enoch et al. looked for sex differences in the influence of COMT Val158Met on alcoholism and smoking in plains American Indians. They found that both male and female alcoholics were more likely to have at least 1 Val158 allele compared with non-alcoholics (0.95 vs. 0.88, *p* < 0.05). Approximately 30% of all participants were long-term, non-addicted light, social smokers (3.6±1.7 cigarettes/d); they had the same Val158Met frequencies as non-smokers (96).

### The opioid receptor genes

Three opioid receptors, mu, delta and kappa exert their pharmacological actions through the *Oprm1, Oprd1*, and *Oprk1* genes, respectively, and these genes have been cloned. A family of natural opiate-like endogenous peptides, (enkephalins, dynorphins, and endorphin), which are released by neurons activate opioid receptors in the brain. The mu (μ) opioid receptors (MOR), located on chromosome 6 at q24-q25, have a high affinity for the enkephalins and beta-endorphin, found in the brain. The prototypical μ-opioid receptor activator is the mu agonist morphine, the primary psychoactive alkaloid in opium. The primary purpose of the mu opiate receptor is the control of pain; it is also very involved in the regulation of dopamine release in the reward site (nucleus accumbens) of the brain. One function of MOR, when activated by either endogenous natural opioids like enkephalins or opioid compounds like Fentanyl or Oxycontin, is to suppress the inhibitory chemical messenger GABA allowing for dopamine release at the reward site. These potent activators of MOR can cause overdose and death by blocking breathing. The risk variant of the MOR is the G allele 118A>G (p.Asn40Asp); SNP rs1799971). The definition of opioid is any synthetic narcotic that has opiate-like activities not derived from opium. The over-prescription of opioids (297 million in 2016) for pain relief, has been linked to the unwanted deaths (one person every 17 min dying from an overdose in America). Studies on the MOR variants are primarily related to substance use disorder: alcohol, food, opiate/opioid, and nicotine dependence; and RDS behaviors: overeating, inability to cope with stress, and PTSD. Carriers of the G allele have a reduced response to opioids (97) and exhibit hypodopaminergia.

Published studies reveal that polymorphisms in the Mu opioid receptor associate with drug dependence. A meta-analysis from Haerian and Haerian is a good example. They sort to resolve the question of whether the OPRM1 rs1799971 polymorphism associated with opioid dependence by evaluating evidence from 13 studies (*n* = 9,385), comprising 4,601 opioid dependents and 4,784 controls. These studies evaluated the association of the OPRM1 rs1799971 polymorphism with susceptibility to opioids. The analysis showed a significant association between this polymorphism and susceptibility to opioid dependence in overall studies under a codominant model, as well as susceptibility to opioid dependence or heroin dependence in Asians under an autosomal dominant model. They concluded OPRM1 rs1799971 might be a risk factor for addiction to opioids or heroin in an Asian population (98).

In another study Marini et al. examined the involvement of the mu-opioid receptor gene polymorphism A118G in the efficacy of detoxification of alcohol-dependent patients. They found that alcohol-dependent patients with the A/A genotype could have a faster restoration of their liver function than those with the A/G genotype: it might be possible that the presence of G allele confers on these patients a reduced ability in abstaining from drinking alcohol (99).

Wang et al. looked at genetic polymorphisms in the OPRM1 gene to determine if in methadone maintenance they are associated with changes in libido and insomnia in patients. The results obtained using dominant model analysis indicate that the OPRM1 SNPs rs1074287, rs6912029, rs12209447, rs510769, rs3798676, rs7748401, rs495491, rs10457090, rs589046, rs3778152, rs563649, and rs2075572 are significantly associated with change-in-libido side effects (adjusted *p* < 0.042). A recessive model analysis, of these SNPs, were found to be significantly associated with insomnia side effects in this cohort (*p* < 0.009). A systematic review and meta-analysis of six previous studies from Chamorro, et al. that analyzed the role of A118G polymorphism in response to naltrexone for AD. After meta-analysis, they found lower relapse rates in patients treated with naltrexone who carried the G allele, than patients who were homozygous for the A allele (OR: 2.02, 95% CI 1.26–3.22; *P* = 0.003) (100).

Bond et al. examined alterations in beta-endorphin binding and activity and the possible implications for opiate addiction. Their results show that SNPs in the mu opioid receptor gene can alter binding and signal transduction in the resulting receptor. This finding may have implications for normal physiology, therapeutics, and vulnerability to develop or be protected from diverse diseases including the addictive diseases. The object of this review is the mu opioid receptor gene, Oprm1 that generates 3 sets of proteins, each containing many variants. The review suggests these variants might be targeted to generate safer, effective analgesic drugs lacking respiratory depression, physical dependence, and reward behavior (101).

### The serotonin transporter gene

The serotonin transporter gene encoded by (SLC6A4) located with the 5-HTTLPR polymorphism on chromosome 17, occurs in the promoter region of the gene. Researchers report two variations in humans; a short (s) and a long (l). The 5-HTTLPR (serotonin-transporter-linked polymorphic region) is a degenerate repeat polymorphic region. There have been thousands of reports many related to behavioral, pharmacogenetic and RDS behaviors since the identification of the polymorphism in the 1990's. The risk variant involves a 43 base –pair 5″ insertion/deletion, S' at SNP rs25531. Researchers found that long allele results in higher serotonin transporter mRNA transcription in human cell lines. Thus, the serotonin is swiftly released into the synaptic cleft; eliminated from the synapse into the pre-nerve cell resulting in low serotonin content in the synapse leading to reduced function. Sometimes the long A allele of SNP rs25531 is written LA. Some studies revealed that the risk form signifies a predisposition to affective disorders, depressive responses to life stress, hyperactivity, and slowing of the electroactivity (speed) of the brain. Carriers of the S' or LA variant are at risk for substance-related: alcohol, opiate/ opioid, nicotine, cocaine, cannabis, and glucose dependence; RDS addictive behaviors: ADHD, PTSD, and pathological gambling and even pain response (102–105).

The established role of the serotonin transporter and associated polymorphisms across the Brain Reward Cascade and addiction liability is exemplified by this study from Herman et al. They examined the association between a measure of sociopathy and 5-HTTLPR genotype in a sample of individuals from a multi-center alcohol treatment trial [Project MATCH]. Regression analysis revealed that males with the L'L' genotype (i.e., those homozygous for the L(A) allele) had lower socialization scores (i.e., greater sociopathy) than males who were carriers of the S' allele (*P* = 0.03). In contrast, women with the S'S' genotype had lower socialization scores than women with two L' alleles (*P* = 0.002) and tended to have lower Socialization Index of the California Psychological Inventory scores than women with one copy of the L' allele (*P* = 0.07). Among individuals with AUDs, the tri-allelic 5-HTTLPR polymorphism had opposite effects on socialization scores in men than women. The basis for this finding is unknown, but it may have implications for sub-typing alcoholics (106). Herman and Balogh explored polymorphisms of the serotonin transporter and receptor genes and susceptibility to substance abuse. They suggested that Genetic variations in the 5-HT system, such as SLC6A4, HTR1B, HTR2A, HTR2C, HTR3 (HTR3A, HTR3B, HTR3C, HTR3D, and HTR3E), likely play a role contributing to SUD patient heterogeneity (107).

Polsinelli, Levitan, and De Luca used a multiple-model meta-analysis to clarify the association between BN and 5-HTTLPR using statistical models not used by previous meta-analyses and extend upon previous meta-analyses by including new samples. Data were pooled using dominant and additive models. Both models showed an association between the 5-HTTLPR polymorphism and BN that was non-significant (108).

Harkness et al. examined the moderating role of childhood emotional, physical and sexual maltreatment and the serotonin-transporter-linked promoter region (5-HTTLPR) polymorphism to stress generation in a cross-sectional community sample of 297 adolescents and young adults. Individuals with the risk s-allele of the serotonin transporter gene and a history of maternal emotional maltreatment or sexual maltreatment reported higher rates of dependent and dependent-interpersonal life events than those homozygous for the l-allele (109).

Liu et al. investigated the relationship between the serotonin transporter gene (SLC6A4) 5-HTTLPR genotypes, cocaine-dependence, and impulsivity in 98 healthy control and 243 treatment-seeking, cocaine-dependent subjects. They found that the impulsivity BIS-11 total score was associated positively with years of cocaine use for S'-allele carriers (*r* = 0.26, *P* = 0.0006, Pearson's correlation analysis), but not for L'L' genotype subjects (*r* = 0.02, *P* = 0.87) (110). It is noteworthy that Garbarino et al. (111), suggested that extreme bidirectional perturbations of serotonin signaling during development of one's DNA likely compound or synergize to facilitate enduring neurochemical changes resulting in insufficient or excessive 5-HT signaling, that could underlie the persistent behavioral characteristics of autism spectrum disorder (111).

### The gamma-aminobutyric acid receptor subunits

GABA a neurotransmitter mediates neuronal inhibition by binding to the GABA/benzodiazepine receptor and opening an integral chloride channel. Gamma-aminobutyric acid receptor subunit alpha-3 (GABRA3) is a human protein that is encoded by the GABRA3 gene. GABA is the major inhibitory neurotransmitter that acts at GABA_A_ receptors to fine-tune dopamine release in the reward site of the brain. Of the 16 identified and distinct subunits of GABA-A receptors, the GABRA3 gene located on Xq28, and the risk variant is CA-Repeat (171-201) whereby allele 181 results in higher activity. This risk allele if overexpressed will cause low dopamine function (hypodopaminergia) leading to SUD: including AD, and other RDS non-substance addictive behaviors and PTSD (112–117).

There are also association studies involving various polymorphisms of GABA receptors and RDS behaviors including alcoholism. An example is an extensive investigation, by Enoch et al. research to identify the functional locus of GABRA2 genotyped 24 SNPs across GABRG1 (gamma subunit) and GABRA2 in 547 Finnish Caucasian men (266 alcoholics), and 311 community-derived Plains Indian men and women (181 alcoholics). GABRG1 haplotypes and SNPs associated significantly with AUD whereas there was no association between AUD and GABRA2 haplotypes. Although of several common (>or = 0.05) haplotypes that spanned GABRG1 and GABRA2 (341 kb) emerged, three of which were present in both populations. One associated with AUD, the other two were more common in non-alcoholics determined by GABRG1. Three less-common extended haplotypes (<0.05) in the Finns, associated with AUD that was determined by GABRA2. These results suggest that there are likely to be independent, complex contributions from both GABRG1 and GABRA2 to alcoholism vulnerability (118).

Terranova et al. analyze the connection between AD and criminal behavior and GABA receptors by an integrated genetic-environmental approach that examined 186 alcohol-dependent males; group 1 (*N* = 47 convicted subjects) compared with group 2 (*N* = 139 no previous criminal records). Genetic results highlighted group 1 differences in genotype distribution (*p* = 0.0067) for SNP rs3780428, found on the intronic region of subunit 2 of the GABA B receptor gene (GABBR2) (119).

Massat et al. (120) found that the GABRA3 polymorphism may confer susceptibility to or may be in linkage disequilibrium with another gene involved in the genetic etiology of bi-polar depression (120). Cui et al. looked at the genetics of GABAergic signaling in nicotine and AD. Human genetic studies support the involvement of genes and variants in the GABAergic signaling system in the etiology of nicotine dependence and alcoholism based on linkage, association, and gene-by-gene interaction studies (121).

Finally, there are multitudes of genetic studies that associate specific behaviors with identified reward gene alleles (specific SNPs) within the mesolimbic pathway. These descriptions of the contribution made by polymorphisms (sequence variations) that effect the healthy function of reward genes, and this small sampling of association studies illustrate why the presence of these alleles in a gene panel indicate genetic risk for associated behaviors.

## Promoting a pro-dopamine lifestyle

A comprehensive treatment program that teaches a pro-dopamine lifestyle and uses urine drug screens like the Comprehensive Analysis of Reported Drugs (CARD) to monitor outcomes, and as a basis for therapeutic interactions, is suggested. Can a pro-dopamine lifestyle with gentle prolonged D2 agonist therapy overcome DNA polymorphisms by promoting positive epigenetic effects which can be transferred from generation to generation (32, 122, 123)? Holistic modalities like exercise (124), low glycemic index diet (125), mindfulness training, neurofeedback, yoga, and meditation are known to support reward neurotramsmission and naturally release dopamine the product of reward neurotransmission (126, 127). These holistic pro dopamine modalities supported by the 12 step fellowship, might induce feelings of well-being and thereby reduce craving and relapse. With this in mind, we wonder if we have been “licking our pups” enough? Could substance and non-substance seeking- behaviors be attenuated through nurturing (128) as suggested by David E. Smith in the late 60's “love needs care” (129)?

## Summary

These basic concepts underpin translational addiction-related research that can help the multitude of victims of genetically induced RDS become the recipients of better therapeutic relapse-preventive tactics.

Finally, as neuroscientists and psychiatrists, working in the “addiction space” we encourage the global scientific community to take heed and reconsider the current utilization of dopaminergic blockade and instead adopt the goal of regaining dopamine homeostasis. Optimistically, early predisposition diagnosis through genetic testing; including pharmacogenetic and pharmacogenomic monitoring, with appropriate urine drug screening, and treatment with pro-dopamine regulators could conceivably reduce stress, craving, and relapse and enhance well-being in the recovery community. Following required basic and clinically directed research, the notion of genetically guided therapy may become a front-line technology with the potential to overcome, in part, the current heightened rates of substance abuse.

## Author contributions

The original concept was developed by KB, DB, JN, PT, and RDB. The original draft was provided by KB and MG-L. The entire paper was carefully vetted by RDB, IE, MG-L, KB, EB, PT, DB, JN, and approved.

### Conflict of interest statement

KB owns stock in some companies holding patents on genetic testing and KB220PAM and is Chairman of the Genus Health LLC scientific advisory board. MG-L, DB, PT, IE, and RDB are members of the Genus Health LLC scientific advisory board. The remaining authors declare that the research was conducted in the absence of any commercial or financial relationships that could be construed as a potential conflict of interest.
